# Commentary: Evolution of *UCP1* Transcriptional Regulatory Elements Across the Mammalian Phylogeny

**DOI:** 10.3389/fphys.2017.00978

**Published:** 2017-11-28

**Authors:** Tobias Fromme

**Affiliations:** Molecular Nutritional Medicine, Else Kröner-Fresenius Center for Nutritional Medicine and ZIEL Institute for Food and Health, Technical University of Munich, Freising, Germany

**Keywords:** uncoupling protein 1, brown adipose tissue, transcriptional regulation, enhancer, evolution, molecular

Adaptive, non-shivering thermogenesis to defend a warm body temperature in a cold environment is provided by brown adipose tissue, a highly specialized organ of endothermic mammals with immense oxidative capacity. On the molecular level of heat production, all processes converge on the essential, thermogenic uncoupling protein 1 (Ucp1), located in the mitochondrial inner membrane (reviewed in Klingenspor and Fromme, 2012). This unique protein is subject to intense investigation in the fields of thermophysiology, energy metabolism and pharmacology.

The comparative study of orthologous Ucp1 gene sequences has been vital to identify the evolutionary origin as well as crucial sites for the regulation of activity and abundance (Klingenspor et al., 2008). Past hallmark findings include the presence of (non-thermogenic) Ucp1 in ectotherm fish (Jastroch et al., 2005, 2007), a rapid gene evolution on the branch leading to Eutherians (Hughes et al., 2009) and the loss of intact Ucp1 in the pig lineage (Berg et al., 2006; Hou et al., 2017). The field of comparative genetic analysis has been steadily gaining momentum with the ever-growing number of fully or partially available genome sequences. In the case of Ucp1, the development peaked this year in comprehensive analyses of more than a 100 amniote (McGaugh and Schwartz, 2017) or mammalian species (Gaudry et al., 2017) providing a framework for all previous findings. The crucial higher-level pattern appears to be a differential pace of Ucp1 evolution since the advent of eutherians: while taxa with small body size continue to race ahead in adapting their thermogenic core component to intense use, other taxa markedly slowed the rate of amino acid exchanges, probably concomitant to an increase in body mass. The reduced importance of brown adipose tissue thermogenesis in large mammals culminates in pseudogenization or complete loss of Ucp1 in pigs, whales and dolphins as well as horses, elephants and sloths. The significance of body mass and thus volume to surface ratio is illustrated by the presence of Ucp1 and brown fat in newborn large mammals and its dramatic loss during the first months of life (Giralt et al., 1989; Soppela et al., 1991). These observations certainly raise the question whether some large species with seemingly intact coding sequence in fact never express the large amounts of Ucp1 protein required for efficient heat production; essentially a pseudogenization event on the level of transcriptional regulation.

Into this context, Gaudry and Campbell place a similarly comprehensive comparison of known Ucp1 regulatory regions, most prominently the distal and complex Ucp1 enhancer (Gaudry and Campbell, 2017).

Conceptually, the validation of regulatory elements of the Ucp1—or any—gene as identified in one species by verifying conservation in orthologous promoters is not a new idea. The absence of a CpG island in the murine Ucp1 promoter and of the complete enhancer in marsupials, for instance, has been discussed before (Jastroch et al., 2008; Shore et al., 2012). The descriptive power of the present study stems from sheer quantity and this statement should not be misunderstood as derogatory. Conversely, “just some more sequences” here turns out to be decisive to detect higher-order patterns in the first place, to then pinpoint individual aberrations worth inspecting closer. Gaudry and Campbell identify a number of regulatory regions with supposedly established function that seem non-essential for efficient Ucp1 expression in many taxa. Eventually, only the proximal TATA box and the well-known distal enhancer region seem to be universal in the control of intact Ucp1 orthologs and exclusively disrupted in pseudogenes.

The diversity of sequences, both regulatory and coding, as collected and presented by Gaudry and coworkers (Gaudry and Campbell, 2017; Gaudry et al., 2017) as well as earlier by McGaugh and Schwartz (2017) can only be described an *El Dorado* for future comparative studies of Ucp1 transcriptional and protein activity regulation. These promise vital insight into both novel options to manipulate Ucp1 expression and activity therapeutically in humans and into ecotype-specific thermoregulatory strategies. The mentioned publications inspire more questions than they answer and compel to re-think future research avenues. It is in fact even a little anticlimactic that Gaudry and Campbell, with all their expertise and amassed sequences, did not themselves choose to continue into some of the more obvious routes and use their *in silico* tools to discover new regulatory and functional elements in promoter and coding sequence instead of simply corroborating or dismissing known ones. For instance, the marsupial Ucp1 gene may be regulated by a different enhancer region than placental mammals (Li et al., 2014).

In future, the collected coding sequences ought to be functionally analyzed in comparable experimental settings (reviewed in Hirschberg et al., 2011) to discover and explore the consequences of ongoing Ucp1 evolution, e.g. the difference between Ucp1 of hibernating hedgehogs and the closely related non-hibernating moles. Functional analyses of regulatory enhancer and promoter sequences may identify taxon-specific expression strategies and their critical elements, e.g., the role of an alternative TATA box sequence in bats and bears and the consequence of an absent CRE-3 element that is extremely well conserved, except in the starmole. Ucp1 expression levels in brown adipose tissue of as many species as possible will detect possible functional pseudogenes with seemingly intact open reading frame. The restoration of Ucp1 expression in species with pseudogenes as already reported for the pig will be an interesting complementary approach to genetic knock-out strategies (Zheng et al., 2017). Furthermore, closely related species with and without expression of functional Ucp1 may prove crucial models to identify Ucp1-independent mechanisms of non-shivering thermogenesis that are suggested by accumulating evidence (Ukropec et al., 2006; Meyer et al., 2010; Bertholet et al., 2017; Keipert et al., 2017; Nyman et al., 2017).

In hindsight, past research on the Ucp1 gene and promoter serve as an apt example how the study of mice and humans may at times mislead into the interpretation of special cases as apparently general insight. It is comprehensive studies comprising many different species, genuinely and confidently descriptive, that allow for the targeted selection of diverse, specialized non-model organisms for informative comparative, physiological studies (von Praun et al., 2001; Jastroch et al., 2007, 2009; Mzilikazi et al., 2007; Trzcionka et al., 2008; Oelkrug et al., 2013; Laursen et al., 2015). It is to be hoped that the large-scale compilations published this year draw more deserved attention to the Ucp1 gene of little studied species that offer superior discovery potential as compared to popular model organisms.

## Author contributions

The author confirms being the sole contributor of this work and approved it for publication.

### Conflict of interest statement

The author declares that the research was conducted in the absence of any commercial or financial relationships that could be construed as a potential conflict of interest.
